# Risk stratification of ER‐positive breast cancer patients: A multi‐institutional validation and outcome study of the Rochester Modified Magee algorithm (RoMMa) and prediction of an Oncotype DX^® ^recurrence score <26

**DOI:** 10.1002/cam4.2323

**Published:** 2019-06-14

**Authors:** Bradley M. Turner, Mary Ann Gimenez‐Sanders, Armen Soukiazian, Andrea C. Breaux, Kristin Skinner, Michelle Shayne, Nyrie Soukiazian, Marilyn Ling, David G. Hicks

**Affiliations:** ^1^ Department of Pathology and Laboratory Medicine University of Rochester Rochester New York; ^2^ Department of Pathology and Laboratory Medicine University of Louisville Louisville Kentucky; ^3^ University of Rochester Rochester New York; ^4^ Department of Surgery University of Rochester Medical Center Rochester New York; ^5^ Department of Medical Oncology University of Rochester Rochester New York; ^6^ Drexel University College of Medicine Graduate School of Biomedical and Professional Studies Philadelphia Pennsylvania; ^7^ Department of Radiation Oncology University of Rochester Rochester New York; ^8^ Department of Pathology and Laboratory Medicine University of Rochester Medical Center Rochester New York

**Keywords:** algorithm, ER^+^ breast cancer, Oncotype DX***^®^***, recurrence, RoMMa

## Abstract

The skyrocketing cost of health‐care demands that we question *when* to use multigene assay testing in the planning of treatment for breast cancer patients. A previously published algorithmic model gave recommendations for which cases to send out for Oncotype DX***^®^*** (ODX) testing. This study is a multi‐institutional validation of that algorithmic model in 620 additional estrogen receptor positive breast cancer cases, with outcome data on 310 cases, named in this study as the Rochester Modified Magee algorithm (RoMMa). RoMMa correctly predicted 85% (140/164) and 100% (17/17) of cases to have a low‐ or high‐risk ODX recurrence score, respectively, consistent with the original publication. Applying our own risk stratification criteria, in patients who received appropriate hormonal therapy, only one of the 45 (2.0%) patients classified as low risk by our original algorithm have been associated with a breast cancer recurrence over 5‐10 years of follow‐up. Eight of 116 (7.0%) patients classified as low risk by ODX have been associated with a breast cancer recurrence with up to 11 years of follow‐up. In addition, 524 of 537 (98%) cases from our total population (n = 903) with an average modified Magee score ≤18 had an ODX recurrence score <26. Patients with an average modified Magee score ≤18 or >30 may not need to be sent out for ODX testing. By avoiding these cases sending out for ODX testing, the potential cost savings to the health‐care system in 2018 are estimated to have been over $100,000,000.

## 1 INTRODUCTION

The evolving era of precision medicine demands both cost‐efficient and cost‐effective strategies for diagnostic treatment algorithms. Challenges remain in accurately assessing which strategies are more cost‐effective for identifying hormone receptor‐positive breast cancer patients who will benefit from systemic chemotherapy.

Over the last decade, molecular approaches, including multigene assays for predicting prognosis and treatment response, have entered into the clinical arena of breast cancer care.^1^, ^2^, ^3^, ^4^, ^5^, ^6^, ^7^, ^8^ All of these multigene assays have been shown to have some prognostic and predictive value in certain subgroups of estrogen receptor (ER) positive breast cancer patients.^3^, ^4^, ^9^ Oncotype DX^®^ (ODX) is the most widely used of these multigene assays. ODX is an expensive test, and although the test has been suggested to be cost‐effective,^10^, ^11^, ^12^, ^13^, ^14^, ^15^, ^16^ it may not be the *most* cost‐effective option in certain subsets of breast cancer patients.^17^, ^18^ A recent meta‐analyses by Wang et al^18^ suggests that a majority of published articles supporting the cost‐effectiveness of ODX were generally industry‐funded, and incorporated study designs that can increase the risk of bias. In a majority of these studies supporting the cost‐effectiveness of ODX, clinical characteristics commonly used to make chemotherapy decisions (ie, tumor size and grade) were not incorporated into simulation modeling. As such, these “supportive” studies might not reflect actual clinical practice.^18^

Several studies have suggested that standard clinical, histopathological, semi‐quantitative immunohistochemistry (IHC), and biomarker data can provide information similar to that provided by the ODX recurrence score (ODXRS).^19^, ^20^, ^21^, ^22^, ^23^, ^24^, ^25^, ^26^, ^27^, ^28^, ^29^, ^30^, ^31^ The IHC4 score^23^ uses semi‐quantitative information from the immunohistochemical assessment of ER, PR, HER‐2, and Ki‐67 (four of the genes measured in the ODX panel) to calculate a risk score using weighting factors and an algorithm. Recent studies have validated the use of the IHC4 score for identifying patients at low, moderate, or high risk of relapse following current endocrine therapy, and the IHC4+ C score (which includes clinical and additional pathologic variables) for identifying patients at low risk who potentially can avoid adjuvant radiotherapy.^32^, ^33^ The equations used for the IHC4 and IHC4+ C scores are available to the public free of charge.^34^

Klein and Dabbs et al^19^ published three linear equations (the new Magee equations) using different combinations of standard histopathological variables (Nottingham score [NS], ER, PR, HER‐2, Ki‐67, and tumor size). These new Magee equations are also available to the public free of charge (https://path.upmc.edu/onlineTools/mageeequations.html), and calculate a recurrence score, which was shown to correlate well with the ODXRS. We published an algorithm^31^ based on a modification of the new Magee equations, showing that this algorithm provides similar risk information to the ODX test.

The goal of this study was to further validate our original algorithm, using data from two separate institutions, and to examine outcome data from two separate institutions. We also present data which suggests further clinical utility of the average modified Magee equation, given the recent TAILORx findings that certain populations of patients with an ODXRS <26 may not benefit from additional systemic chemotherapy.^35^

## 2 MATERIALS AND METHODS

### 2.1 Patients and data retrieval

**Figure 1:**
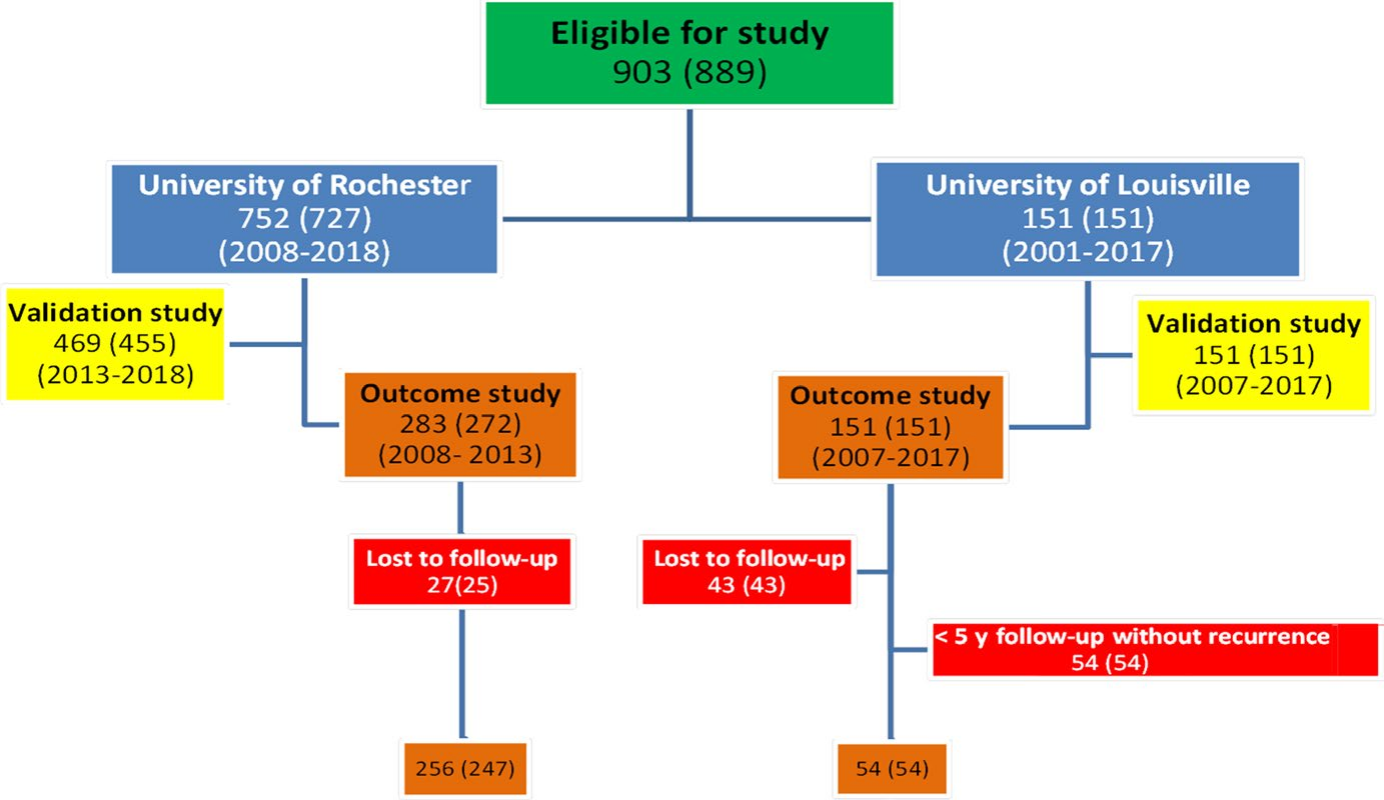
Number of cases (patients) used for the validation and outcome evaluations

**Figure 2:**
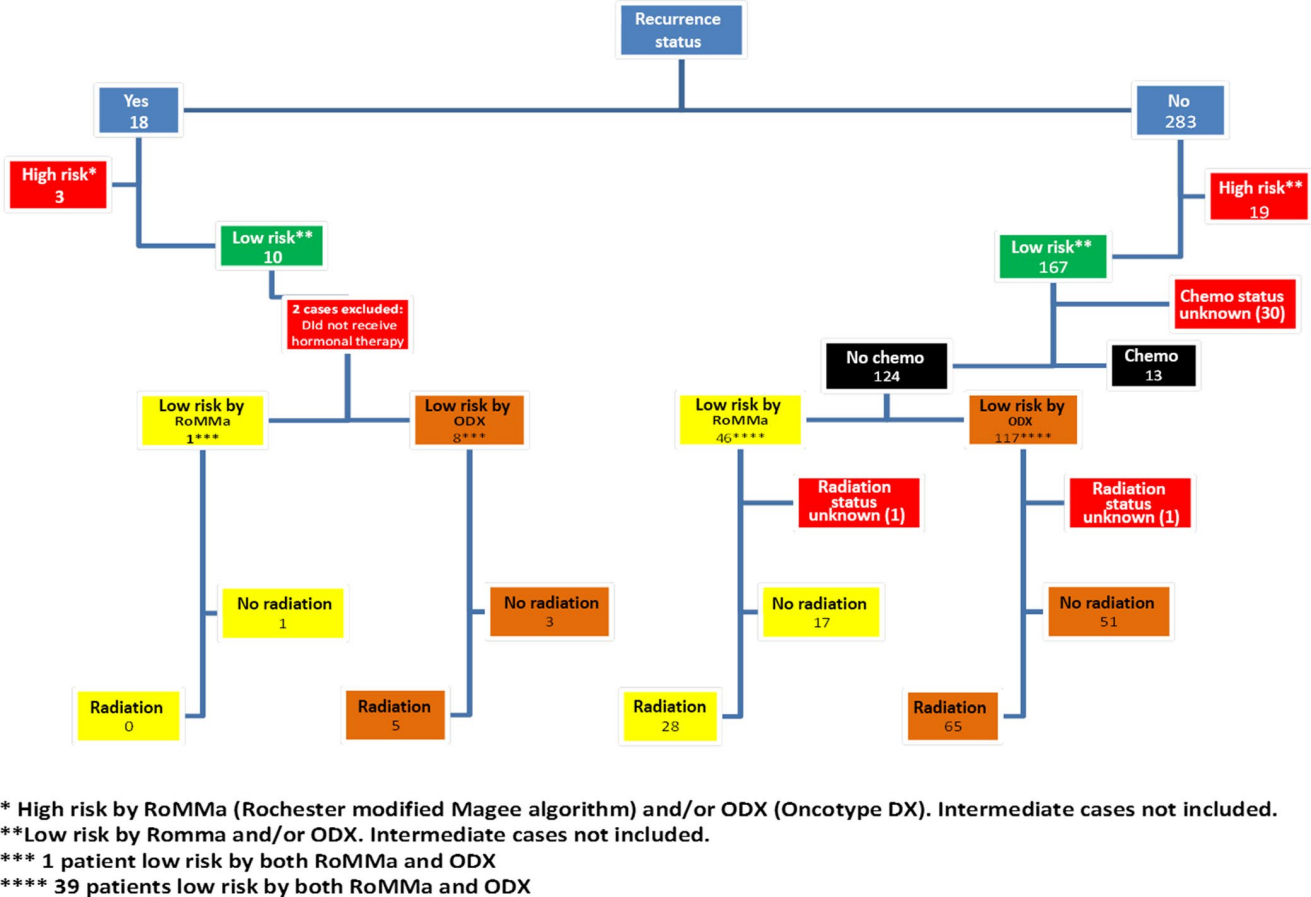
Patients used for the outcome evaluations

A total of 903 consecutive cases (889 patients) with ER^+^ invasive breast cancer were included in this study from the University of Rochester and the University of Louisville. Figures 1 and 2 highlight the cases that were used for the validation and outcome evaluations. All 903 cases were used for the evaluation of an ODXRS <26.

Six hundred and twenty of 903 cases (606/889 patients) were included in the validation study (Figure 1). None of these cases were used in our original 2015 publication.^31^

Three hundred and ten of 903 cases (301/889 patients) were included in the outcome analysis (Figures 1 and 2). The outcome analysis included all patients who had at least 5 years of follow‐up data, and all patients who had a breast cancer recurrence.

Information on ER, PR, HER‐2, Ki‐67, and tumor size was extracted from the pathology report. The NS was calculated using the Nottingham modification of the Bloom‐Richardson system.^36^ Information on age, ethnicity, lymph node status, lymphovascular invasion, hormone therapy, chemotherapy, radiation therapy, recurrence status, and mortality were extracted from the medical record. Some type of hormonal therapy or therapies (Anastrozole, Exemestane, Tamoxifen, Lupron, and/or Letrozole) was known to have been received in 240 patients. Some type of systemic chemotherapy or therapies (Carboplatin, Cyclophosphamide, Docetaxel, Doxorubicin, 5‐flourouracil, Methotrexate, Paclitaxel, and/or Vinorelbine) was known to have been received in 59 patients. Some type of radiation therapy was known to have been received in 158 patients. All tumor H&E slides and IHC were reviewed by at least two board‐certified breast pathologists, with manual interpretation of ER, PR, HER‐2, and Ki‐67, using standard histological criteria for determining modified ER and PR H‐scores^31^ (FDA‐approved test kits [DAKO] ‐ ERα [clones ID5 and ER‐2‐123]; PR [clone PgR1294] pharmDxTM), and HER‐2 IHC scores (Rabbit anti‐human HER‐2 HercepTestTM). HER‐2 FISH was performed (FDA‐approved test kit [DAKO] ‐ HER‐2 IQFISH pharmDxTM) on all equivocal HER‐2 IHC results, and the FISH results were used in lieu of the IHC for these cases. Ki‐67 was evaluated by calculating the percentage of positive staining tumor cells on a single slide (Monoclonal mouse anti‐human Ki‐67 antigens [clone MIB‐1, code M7240]).

### 2.2 Study design

The algorithmic approach used has been previously described by Turner et al^31^ Briefly, an average modified Magee recurrence score was calculated, and all cases with an average modified Magee recurrence score of ≤12, or with a modified ER and PR H‐score ≥150 and a Ki‐67 <10%, were considered low risk. All cases with an average modified Magee recurrence score >30 were considered high risk. We compared our current results from the validation study with our results from the 2015 publication. We also examined different average modified Magee recurrence score groups (ie, groups with a score ≤9, groups with a score of 10, groups with a score of 11, etc) and their associated‐average ODXRS. Finally, we examined the association of breast cancer recurrence outcome data with the average modified Magee recurrence score, ODXRS, and clinicopathological data.

### 2.3 Statistical analysis

Available clinical and pathological data were summarized using percentages, descriptive statistics (mean, range, frequencies), and inferential statistics (chi‐square (χ^2^) test of independence, Pearson correlation, odds ratio, and t‐test). Each patients average modified Magee score was compared to their ODXRS using the ODXRS risk stratification categories [low (<18), intermediate (18‐30), or high (>30)]. All data analyses were performed using the statistical Analysis ToolPak (Microsoft Excel Office 2010 version 14.0.7015.100) except for the odds ratios and chi‐square (χ^2^) test of independence, which were performed with JavaStat 2‐way Contingency Table Analysis (revised version 7/23/2013 http://statpages.org/ctab2x2.html). For all results, a *P*‐value of <0.05 was considered significant. This study received IRB approval from the University of Rochester (IRB# RSRB00069270).

## 3 RESULTS

A summary of clinicopathological features in the patient population is detailed in Tables 1 and 2.

**Table 1:** Demographic characteristics of validation cases^a^

Mean age, years (range), n = 606	59.1 (28‐85)	
	N (%)	
	Patients^b^	Cases
All	606 (100)^c^	620 (100)^c^
Ethnicity		
White	534 (88.1)	547 (88.2)
African American	50 (8.3)	51 (8.2)
Hispanic	8 (1.3)	8 (1.3)
Asian	8 (1.3)	8 (1.3)
Unknown	6 (1.0)	6 (1.0)
Average modified Magee score^d^		
Score ≤9 (low risk)	12 (2.0)	12 (1.9)
Score ≤12 (low risk)	94 (15.5)	97 (15.6)
Score >30 (high risk)	17 (2.8)	17 (2.7)
Nottingham score < 6; Modified ER/ PR H‐score ≥ 150;Ki‐67 < 10% (low risk)^e^	64 (10.6)	67 (10.8)
Oncotype DX<sup>®</sup> recurrence score		
<11 (Oncotype DX^®^ low risk)	131 (21.6)	134 (21.6)
11‐17 (Oncotype DX^®^ low risk)	217 (35.8)	218 (35.2)
18‐30 (Oncotype DX^®^ intermediate risk)	209 (34.5)	210 (33.9)
<26 (TAILORx lower risk group)	478 (78.9)	491 (79.2)
>30 (Oncotype DX^®^ high risk)	58 (9.6)	58 (9.4)
Lymph Nodes		
Negative^f^	468 (77.2)	477 (76.9)
Positive	94 (15.5)	95 (15.3)
Unknown	46 (7.6)	48 (7.8)
Available Nottingham score	606 (100)	620 (100)
Available ER‐H‐score	606 (100)	620 (100)
Available PR H‐score	606 (100)	620 (100)
Available Ki‐67	570 (94.1)	584 (94.2)
Available tumor size	604 (99.7)	618 (99.7)
Available Her‐2 IHC	606 (100)	620 (100)
0+	175 (28.9)	180 (29.0)
1+	268 (44.2)	274 (44.2)
2+	165 (27.2)	165 (26.6)
3+	1 (0.2)	1 (0.2)
FISH	171 (28.0)	171 (27.6)

a Analyses of age, ethnicity, lymph node status, lymphovascular invasion, and years of follow‐up, include all *patients*. Analysis of average modified Magee score, ODXRS, NS, modified ER H‐score, modified PR H‐score, Ki‐67, tumor size, HER‐2 IHC, and HER‐2 fluorescence in situ hybridization (FISH), includes all case data.

b May not equal 100%, as several patients have multiple tumors which fall into different risk stratification groups, and not all risk stratification groups are evaluated for the average modified Magee score.

c 469 cases from 455 different patients (393 invasive ductal carcinomas and 76 invasive lobular carcinomas) were identified from the pathology files at the University of Rochester Medical Center. Fourteen patients had two different tumors from two separate sites. 151 cases from 151 different patients (128 invasive ductal carcinomas and 23 invasive lobular carcinomas) were identified from the pathology files at the University of Louisville.

d Average modified Magee scores >12 and ≤30 that do not meet low risk histologic criteria comprise the intermediate group (n = 478 [78.9%] patients, 487 [78.5%] cases). These cases would be recommended for Oncotype DX^®^ testing.

e Forty‐eight cases (47 patients) with an average modified Magee score ≤12 and meeting low risk histologic criteria; Nineteen cases (17 patients) with an average modified Magee score >12, but meeting low risk histologic criteria.

f Includes patients with isolated tumor cells (n = 10).

**Table 2:** Demographic data on outcome cases^a^

Mean age, years (range), n = 301			57.2 (21‐84)
	N (%)		Mean years of follow‐up (range)
	Patients^b^	Cases	
	301 (100)^c^	310 (100)^c^	6.6 (2‐11)
RoMMa risk stratification^d^			
Average modified Magee score ≤ 9 (low risk)	13 (4.3)	13 (4.2)	**7.4 (5‐10)**
Average modified Magee score ≤ 12 (low risk)	55 (18.3)	57 (18.4)	**6.9 (5‐10)**
Average modified Magee score > 30 (high risk)	12 (4.0)	12 (3.9)	**6.3 (3‐9)**
Nottingham score < 6; Modified ER/ PR H‐score ≥ 150;Ki‐67 < 10% (low risk)^e^	38 (12.6)	39 (12.6)	**6.7 (5‐9)**
Oncotype DX® recurrence score			
<11 (Oncotype DX^®^ low risk)	50 (16.6)	52 (16.8)	**6.8 (5‐11)**
11‐17 (Oncotype DX^®^ low risk)	119 (39.5)	121 (39.0)	**6.7 (2‐11)**
18‐30 (Oncotype DX^®^ intermediate risk)	110 (36.5)	111 (35.8)	**6.6 (2‐10)**
<26 (TAILORx lower risk group)	258 (85.7)	266 (85.8)	**6.7 (2‐11)**
>30 (Oncotype DX^®^ high risk)	24 (8.0)	24 (7.7)	**6.0 (2‐10)**
Lymph node status			
Negative^f^	236 (78.4)	244 (78.7)	**6.7 (2‐11)**
Positive	52 (17.3)	53 (17.1)	**6.0 (2‐10)**
Unknown	13 (4.3)	13 (4.2)	**7.3 (5‐10)**
Lymphovascular invasion status^g^			
Identified	35 (11.9)	35 (11.6)	**6.2 (2‐10)**
Not identified	**258 (88.1)**	**267 (88.4)**	**6.7 (3‐11** **)**

a Analyses of age, lymph node status, lymphovascular invasion, recurrence status, and years of follow‐up, include all *patients*. Analysis of average modified Magee score, ODXRS, NS, modified ER H‐score, modified PR H‐score, and Ki‐67 includes all case data.

b May not equal 100%, as several patients have multiple tumors which fall into different risk stratification groups.

c Nine patients had two different tumors from two separate sites.

d Rochester Modified Magee Algorithm. Average modified Magee scores >12 and ≤30 that do not meet low risk histologic criteria comprise the intermediate group (n = 222 [73.8%] patients, 229 [73.9%] cases). These cases would be recommended for Oncotype DX^® ^testing.

e Twenty‐seven cases (26 patients) with an average modified Magee score ≤12 and meeting low risk histologic criteria; Twelve cases/patients with an average modified Magee score >12, but meeting low risk histologic criteria.

f Includes isolated tumor cells (n = 7).

g n = 293 patients, 302 cases; Not included ‐ four “suspicious” cases [mean years of follow up 5.5 (5‐6)], four unknown cases [mean years of follow up 6.5 (4‐10)].

### 3.1 Correlation and concordance between original publication and the validation population

**Figure 3:**
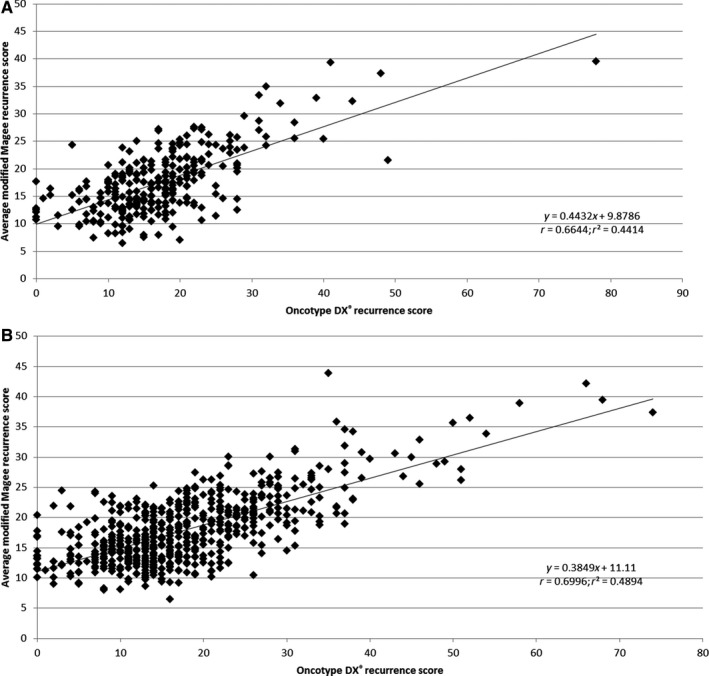
A, Correlation of average modified Magee recurrence score and Oncotype DX^®^ recurrence score from the original publication.^31^ B, Correlation of average modified Magee recurrence score and Oncotype DX^®^ recurrence score in the validation population

Overall, the data in our validation population were remarkably consistent with the data in our original test population from the original publication^31^ (Figure 3A,B, Table 3, and Table S1). A Pearson correlation shows a significant correlation (*P* < 0.0001) between the original test population and the validation population when comparing the percentage of cases with each Magee score in the two groups (Figure S1 and Table S2). A Pearson correlation also shows a significant correlation (*P* < 0.0001) between the original test population and the validation population when comparing the percentage of cases in both a particular Magee score group and its correlating ODX risk group (Figure S2 Table 3 and Table S3).The chi‐squared (χ^2^) test of independence showed no significant difference between the observed frequencies in the original test population and the validation population (*P* = 0.351) when evaluating the number of cases with an average modified Magee score <18 or ≥18 (Table S4). The chi‐squared (χ^ 2^) test of independence also showed no significant difference between the observed frequencies when evaluating cases with an average modified Magee score ≤18 in the original test population and the validation population (*P* = 0.559), that have an Oncotype DX score <26, or ≥26 (Table S5).

**Table 3:** Average modified Magee recurrence score groups and associated Oncotype DX^®^ risk categories from the validation population (n = 620) and original test population^31^ (n = 283)

Average modified Magee score (amMs)^a^	Oncotype DX^®^ recurrence score Validation population				Oncotype DX^® ^recurrence score Original population			
	High	Intermediate	Low	%^b^	High	Intermediate	Low	%^b^
amMs <9	0	0	4	100.0%	0	0	5	100.0%
amMs ≤10	0	2	22	91.7%	0	2	21	91.3%
amMs ≤11	0	7	45	86.5%	0	5	31	86.1%
amMs ≤12	0	13	84	86.6%	0	9	43	82.7%
amMs ≤14	0	22	125	85.0%	0	12	61	83.6%
amMs ≤15	1	36	206	84.8%	0	26	96	78.7%
amMs <18	1	63	261	80.3%	0	42	116	73.4%
NS <6 and ER/PR ≥150 AND Ki67 <10%	0	11	56	83.6%	0	4	34	89.5%
amMs >30	17	0	0	100.0%	8	0	0	100.0%

a Cases with an available Ki‐67.

b Percent Oncotype DX^®^ low risk except for amMs >30 which would be percent Oncotype DX^®^ high risk.

### 3.2 Cases with an average modified Magee recurrence score ≤18 and >18

Five hundred and twenty four of 537 (98%) cases with an average modified Magee score ≤18 had an ODXRS <26 (Table 4). One hundred and thirty three of 366 (36%) cases with an average modified Magee score >18 had an ODXRS ≥26 (Table 4). One hundred and thirty three of 146 (91%) cases with an ODXRS ≥26 had an average modified Magee score >18 (Table 4). An average modified Magee score is highly specific and predictive for an ODXRS <26 (Table 4).

**Table 4:** Average modified Magee recurrence scores ≤18 and >18 and corresponding Oncotype DX^®^ recurrence scores <26 and ≥26 (n = 903)

Average modified Magee score	Oncotype DX^®^ recurrence score Total population (n = 903)	
	<26	≥26
≤18	524	13
>18	233	133

Sensitivity: 0.692.

Specificity: 0.911.

Positive predictive value: 0.976.

Negative predictive value: 0.363.

Odds ratio: 23.0 (*P* < 0.0001).

### 3.3 Outcome analysis

Eighteen of 301 (6%) patients in our outcome population have had a breast cancer recurrence (supplemental Table S6). Overall, two of 66 (3%) patients classified as low risk by our original algorithm recurred, and 10 of 156 (6%) patients classified as low risk by ODX recurred (Table 5). Two of the 18 patients who recurred did not receive hormonal therapy and these two patients were not included in our subsequent analysis of recurrence in “low risk” patients (Figure 2). Considering all the low risk patients who *did* recur except for the two that did not receive hormonal therapy (Table S6), and only the low risk patients who *did not* recur and who *did not* receive chemotherapy (Figure 2), none of the 28 (0%) patients recurred who were classified as low risk by our original algorithm who received radiation, five of the 65 (8%) patients recurred who were classified as low risk by ODX who received radiation, one of the 17 (6%) patients recurred who were classified as low risk by our original algorithm who did not receive radiation, and three of the 51 (6%) patients recurred who were classified as low risk by ODX who did not receive radiation. Seventeen of 18 (94%) patients who recurred had an average modified Magee score ≥13.5, and 13 of 18 (72%) patients who recurred had an average modified Magee score >18 (Table S6).

**Table 5:** RoMMa risk stratification, Oncotype DX^®^ risk stratification, and recurrence

	Recurrence	
RoMMa risk stratification	Yes n (%)	No n (%)
Average Magee score ≤12 (low risk)	1 (2)	54 (98)
NS <6; Modified ER/ PR H‐score ≥150;Ki‐67 <10% (low risk)^a^	1(3)	37(97)
Oncotype DX® recurrence score		
<11 (Oncotype DX^®^ low risk)	1 (2)	48 (98)
11‐17 (Oncotype DX^®^ low risk)	9 (8)	108 (92)
18‐30 (Oncotype DX^®^ intermediate risk)	5 (5)	107 (95)
<26 (TAILORx lower risk group)	12(5)	246 (95)
>30 (Oncotype DX^®^ high risk)	3 (13)	20(87)

a Twenty‐seven cases (26 patients) with modified Magee score ≤12 and meeting low risk RoMMa histologic criteria; Twelve cases/patients with modified Magee score >12, but meeting low risk RoMMa histologic criteria.

Patients who recurred had a significantly higher Ki‐67 (*P* < 0.0001) than patients who did not recur (Table S7). Overall, patients who recurred had a lower PR status (*P* = 0.12), statistically significant in patients with positive lymph nodes (*P* = 0.02) and in patients who did not receive chemotherapy (*P* = 0.02) (Table S7). Overall, neither NS (*P* = 0.45) nor ER status (*P* = 0.54) were significantly different between patients who did and did not recur (Table S7).

Patients who recurred had a significantly higher risk (OR = 6.2, *P* = 0.002) for having LVI (7/17[41%], supplemental Table S7) compared to patients who did not recur (28/276 [10%]). Patients who recurred also had a higher risk (OR = 2.0, *P* = 0.175) for LN involvement (5/18 [28%]) compared to patients who did not recur (47/270 [17%], Table S7), although this did not reach statistical significance.

All the patients with a known Ki‐67 who recurred (n = 13, Table S6) had some combination of a lowered modified PR H‐score (≤ 210), LN involvement, LVI, or a higher Ki‐67 (≥20). Twelve of the 18 patients who recurred in our population (67%) had an ODXRS of <26 (Table 5 and Table S6). Eleven of these 12 patients (92%) were 50 years or older at the time of diagnosis (Table S6).

### 3.4 Cost analysis

**Figure 4:**
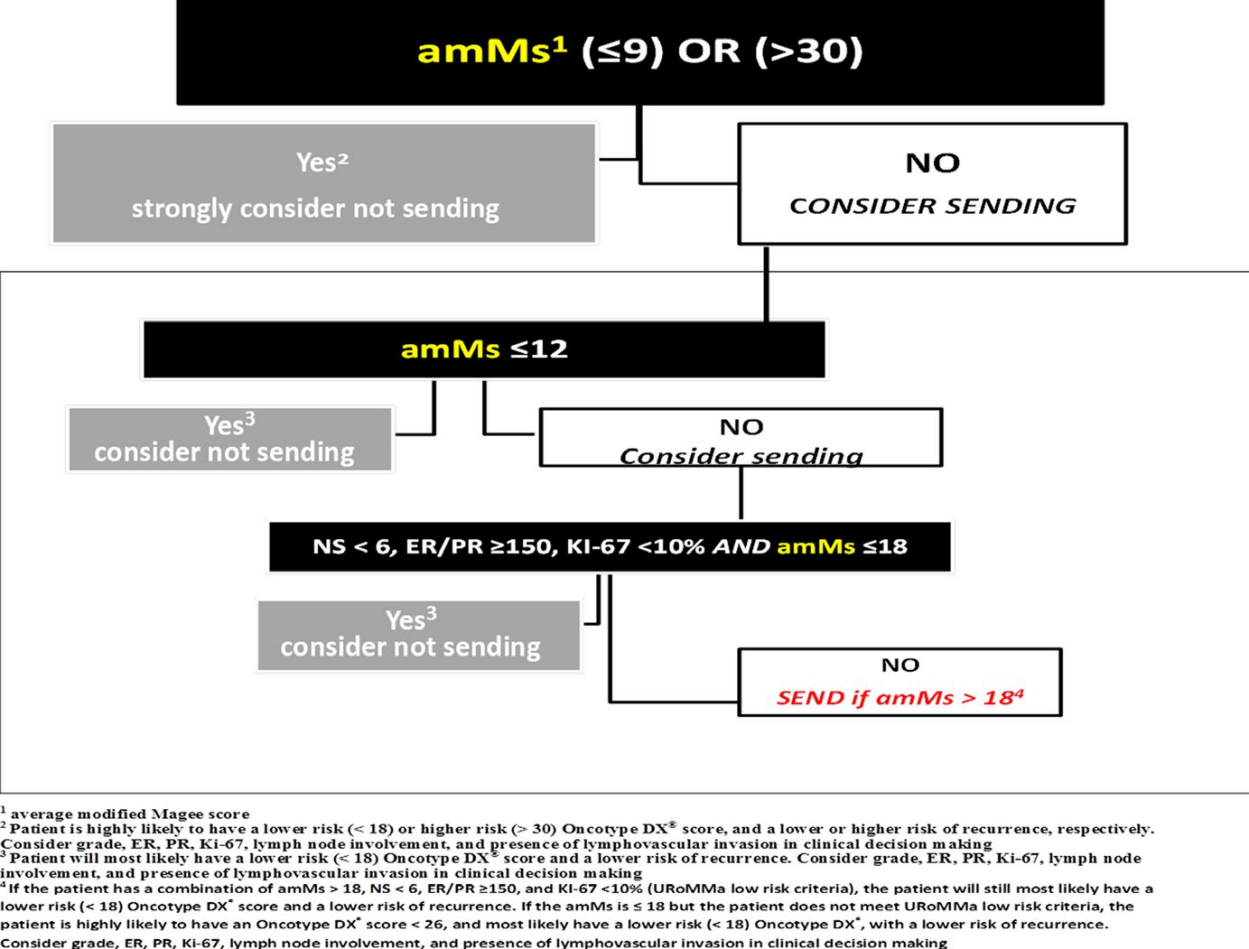
Rochester Modified Magee algorithm (RoMMa)

The list price reported in the Genomic Health 2017 Annual Report^37^ for the invasive breast carcinoma ODX test was $4,620. Using our previously published^31^ algorithmic approach (Figure 4) in our total population of cases between 2007 and 2018 (n = 903), 20.8% (n = 188) satisfied low risk algorithmic criteria, and 2.8% (n = 25) satisfied high risk algorithmic criteria. These 213 cases would potentially not have been sent out by our institutions for ODX testing, creating a combined potential cost savings of $984,060 for the University of Rochester and the University of Louisville. In that same 2017 annual report, “more than 126,740 ODX test reports were delivered for use for treatment planning.”^37^ The substantial majority (approximately 85%) of historical revenues from ODX testing comes from invasive breast carcinoma testing,^31^, ^37^, ^38^ suggesting that approximately 107,729 ER^+^ invasive breast cases were received for ODX testing in 2017. The ODX risk stratification of these 107,729 breast cancer cases was not available for this study; however, similar to our previous publication,^31^ we can envision a reasonable cost‐containment scenario. If we assume that the percentage of algorithmic low risk (20.8%) and high risk (2.5%) cases in our study is similar to the percentage of algorithmic low and high risk cases sent out for ODX testing, then approximately 25,424 breast cancer cases would potentially have been considered as cases not to send out for additional testing with ODX, resulting in an estimated cost savings to the health‐care system in 2018 of $117,459,083.

## 4 DISCUSSION

At the turn of the 21st century, Perou^39^ and Sorlie^40^ reported four significant groups of breast cancer subtypes using RNA expression profiling: luminal A (ER‐positive, HER‐2 negative), luminal B (ER‐positive, HER‐2 positive), HER2‐overexpressing (ER and PR negative, HER‐2 positive), and basal‐like. Prat et al^41^ later included a subtype of basal‐like breast cancer termed claudin‐low breast cancer. Basal‐like and claudin‐low breast cancers are predominantly ER PR and HER‐2 negative, or triple negative (TN). Compared to the luminal A subtype, women with luminal B tumors and HER‐2 overexpressing breast carcinoma have roughly a twofold increased adjusted risk of breast cancer mortality,^42^ and women with triple negative breast cancers have a poorer short‐term prognosis than all the other subtypes.^43^, ^44^ Hormonal therapy with systemic chemotherapy and targeted therapy with monoclonal antibodies (traztuzamab with or without pertuzamab) is the primary established treatment for patients with luminal B breast carcinoma; systemic chemotherapy and targeted therapy with monoclonal antibodies (traztuzamab with or without pertuzamab) is the primary established treatment for patients with HER‐2 overexpressing breast carcinoma; and, systemic chemotherapy is the primary established treatment for patients with TN breast carcinomas. Hormonal therapy is the primary established treatment for patients with luminal A breast carcinoma; however, a subset of luminal A breast carcinoma patients will also benefit from systemic chemotherapy. Challenges remain in accurately identifying which strategies are more cost‐effective and more cost‐efficient in identifying this unique subset of luminal A breast carcinoma patients who will also benefit from systemic chemotherapy.

While molecular approaches, including multigene assays, have been shown to have some prognostic and predictive value in certain subgroups of ER^+^ breast cancer patients,^3^, ^4^, ^9^ standard clinical practice has paid little attention to the suitability of breast tissue for molecular analysis, and current research suggests that alterations in the molecular integrity of breast tissue during the pre‐analytic stage may result in inaccurate results and potentially sub‐standard patient care.^45^ With so many new molecular assays available, on a case by case basis, there remains significant uncertainty on the part of many clinicians on how to best utilize this new molecular information, what incremental value these tests provide, or how best to integrate these assay results with the available clinicopathologic features of the patient's tumor.^17^, ^18^

ODX in particular is of considerable interest. Interestingly, four of the 16 cancer‐related genes measured by ODX (ER, PR, HER2, and Ki‐67) are routinely assessed by IHC as part of the diagnostic evaluation of breast cancer. Kim et al have recently proposed a model that accurately predicts ODX high‐ and low‐risk categories using Elston grade, ER, PR, HER‐2, and Ki‐67.^46^ Several studies have shown that linear regression equations which incorporate histopathological data, ER, PR, HER‐2, and Ki‐67, can also provide information that can be used to predict the ODXRS with a high degree of accuracy.^19^, ^30^, ^31^ We previously published data supporting the use of linear regression equations to risk stratify patients into low and high risks of recurrence,^31^, ^47^, ^48^ introducing an algorithmic approach using the modified Magee Equation 31 Hou et al have recently published data supporting our original study conclusions.^49^, ^50^ We now have additional data validating our original algorithm, which we are now calling the Rochester Modified Magee algorithm (RoMMa, Figure 4). Our validation study supports that our algorithmic approach is a cost‐effective, cost‐efficient alternative to ODX in risk stratifying certain breast cancer patients.

Consistent with our previously published data, in the current validation population, all the patients with an average modified Magee recurrence score >30 (n = 17), or an average modified Magee recurrence score <9 with an available Ki‐67 (n = 4), were correctly predicted to have a high or low ODXRS, respectively (Table 3). Our current results on a separate population of patients from two different institutions give further validation that our algorithm can be used by the clinician when considering which cases not to be sent out for ODX testing.

In our previous study^31^ there was no ‘two‐step’ discordance (a discordant high and low recurrence score *using the ODX risk stratification criteria* between a patients' average modified Magee recurrence score and ODXRS). There was a single case in our validation study with ‘two‐step’ discordance (Table S1). This case was high grade (NS = 8), with a Ki‐67 of 15%, and an average modified Magee score of 15.4. The ODXRS was 31. Using the RoMMa algorithmic risk stratification criteria (*not the ODX risk stratification criteria*), this case would have been RoMMa intermediate (and would have reflexed for ODX testing).

We examined the available outcome data of 301 patients from the original study (Table 2). In the original publication,^31^ 11 patients with available follow‐up had an average modified Magee recurrence score ≤9 (with or without a Ki‐67). Nine of these 11 patients had a low ODX score. The other two patients had an intermediate ODX score of 19 and 20. None of these 11 patients have had a breast cancer recurrence. In the current validation population all the patients with an average modified Magee recurrence score ≤9 (n = 12) were correctly predicted to have a low ODXRS. As such, the original algorithm^31^ is slightly modified in that we consider patients who have an average modified Magee recurrence score ≤9 to be lowest risk (as opposed to just patients with an average modified Magee recurrence score < 9).

Bhargava et al found that 141 of 144 (98%) cases with new Magee equation scores < 18, or new Magee equation scores 18‐25 and mitosis score of 1, had an ODXRS < 26.^51^ Our data using the average modified Magee equation in larger population support these findings. In our population, 524 of 537 (98%) cases with an average modified Magee score ≤18 had an ODXRS <26 (Table 4). Based on this finding, we additionally modified the original algorithm to include consideration for an average modified Magee score ≤18 as “low risk” given the recent TAILORx findings.^35^

Sixty seven percent (12/18) of patients who recurred in our population had an ODXRS of <26 (Table 5 and Table S6). Eleven of these 12 patients (92%) were 50 years of age or older at the time of diagnosis. Three of these 11 patients did not receive any hormonal treatment, and only two of these 11 patients received any adjuvant systemic chemotherapy. The recent TAILORx results suggest that women with early breast cancer who are older than 50 years with an ODXRS <26 can be spared adjuvant chemotherapy.^35^ Our findings suggest that risk stratification with consideration for systemic chemotherapy is still important in these patients.

Our outcome data support that patients designated as low risk by our original algorithm (average modified Magee score of ≤12 or a combination of NS <6, ER/PR ≥150, and KI‐67 <10%) are unlikely to recur. Considering the population of low risk patients who recurred after receiving appropriate hormonal therapy and the population of low risk patients who did not recur and did not receive chemotherapy, one of the 45 (2%) patients classified as low risk by our original algorithm recurred, compared to eight of 116 (7%) patients classified as low risk by ODX who recurred (Figure 2).

Ninety four percent (17/18) of the patients who recurred had an average modified Magee score ≥13.5 (Table S6). Patients who recurred were more likely to have a lower PR, a higher Ki‐67, LN involvement, and LVI than were patients who did not recur (Table S7). Interestingly, neither the average NS nor ER status was significantly different in the recurrence and non‐recurrence populations (Table S7). Thirteen of the 18 patients who recurred had some *combination* of a lowered PR, a higher Ki‐67, LN involvement, or LVI (Table S6). In the five other patients who recurred (patient #1, #5, #8, #9, and #11), either the Ki‐67 and/or the LVI status was unknown. Our results support that the average modified Magee score, PR, Ki‐67, LN, and LVI status may be helpful in predicting patients with a higher risk of recurrence, and should be considered when risk stratifying breast cancer patients for systemic chemotherapy.

ODX testing was rapidly adopted into clinical care of breast cancer patients in 2004 without any randomized trials, based on small cohort studies which suggested that the ODXRS influenced patient preference and oncologist recommendations for chemotherapy.^52^ Although a number of studies have suggested that ODX is cost‐effective, with the cost of the test being offset primarily by reduction in the use of adjuvant chemotherapy,^10^, ^11^, ^12^, ^13^, ^14^, ^15^, ^16^, ^52^ these “supportive” studies have generally been industry funded, not based on real‐world population data, and may not reflect actual clinical practice.^18^, ^52^ A recent study based on real‐world population based data by Mittmann et al suggests that using the ODXRS to determine whether adjuvant chemotherapy should be added to endocrine therapy in ER^+^ lymph node‐negative breast cancer patients was approximately $3,000 more expensive per patient than not using the test.^52^ Our results suggest a potential estimated cost savings to the health‐care system in 2018 of over $100,000,000 if ODX testing was avoided in certain low‐ and high‐risk RoMMa patients. If all cases with an average Modified Magee score of ≤18 were also considered, the cost savings would undoubtedly be substantially higher.

The reproducibility and accuracy of histological grading and immunohistochemical reporting creates a concern for interpretation bias. The literature supports the reproducibility of ER, PR, HER‐2,^53^, ^54^, ^55^, ^56^, ^57^ and Ki‐67,^58^ if standardized criteria are used for their interpretation. We used standard criteria for the evaluation of ER, PR, HER‐2, and Ki‐67, and there was similar agreement in our study between the two institutions in the interpretation of the average modified Magee score relative to the ODX score (Table S1).

All of the patients without recurrence in our population had at least 5 years of follow‐up; however, only 16 of 301 (5%) of patients had 10 or more years of follow‐up. Although 5‐year follow‐up data are acceptable for evaluation of outcome in the published literature, 10 years or more of follow‐up is preferable. We continue to maintain our database of patients, with the goal of additional outcome evaluation that has 10 or more years of follow‐up. Access to larger database populations, such as ECOG, NSABP B‐14, NSABP B‐20, and SEER‐Medicare, would be helpful in providing additional longitudinal data with 10 or more years of follow‐up, which we believe will further validate our findings.

It is likely that variations in adherence to hormonal, systemic, and radiation therapies occurred within our population. A prospective study design or access to data obtained in a prospective fashion would help to eliminate this bias.

The 8th edition of the American Joint Commission on Cancer (AJCC) Cancer Staging manual^59^ determined it was appropriate to incorporate the ODXRS into staging for the subgroup of invasive breast carcinoma patients defined by Arm A of the TAILORx study,^60^ which includes ER^+^, HER‐2 negative, LN‐negative tumors that are 1.1‐5.0 cm in size (or 0.6‐1.0 cm with intermediate or high histologic or nuclear grade), and have an ODXRS <11. According to the AJCC recommendations, these patients should be placed into the same prognostic category as patients with pT1‐ N0 M0 (stage IA) breast cancers (AJCC Prognostic Stage Group I). Our results suggest that the likelihood of breast cancer recurrence with an average modified Magee score ≤12 is comparable with an ODXRS <11 (Table 5). One of the 49 (2%) patients with an ODXRS <11 recurred (Table 5 and Table S6). One of the 55 (2%) patients with an average modified Magee score ≤12 recurred, and this patient did not receive hormonal therapy (Table 5 and Table S6). However, neither RoMMa nor the average modified Magee score can reliably predict an ODXRS <11. It is worth noting, however, that *ODX testing is not a requirement for staging*, and Breaux et al^61^ have shown that an ODX score <11 changes the stage in only rare cases.

The large cost savings in our study is due to the assumption that none of the patients meeting low or high risk RoMMa criteria would receive ODX testing. It is likely that a number of these patients would still receive ODX testing based on many factors, not the least of these being patient and clinician concerns about not using a more accepted (and publicized) test; however, our study highlights the importance of considering other valid and less costly methods for assessing the risk of breast cancer recurrence. Additional studies testing the cost‐efficiency and cost‐effectiveness of integrating the RoMMa into clinical practice are necessary.

## 5 CONCLUSIONS

Our validation results continue to support that patients who satisfy our algorithmic low‐risk or high‐risk criteria are likely to have a low‐risk or high‐risk ODX, respectively.

Specifically, patients with an average modified Magee score of ≤9 are highly likely to be low risk by ODX criteria. Patients with an average modified Magee score ≤12 or, a combination of an average modified Magee score ≤18 with a NS < 6, ER/PR ≥150, and KI‐67 <10% (RoMMa low risk histologic criteria), are also very likely to be lower risk by ODX criteria. Patients with an average modified Magee score of >30 are highly likely to be high risk by ODX criteria. Consideration should be given to not sending out tissue for ODX testing in patients meeting low‐ or high‐risk RoMMa criteria, if treatment decisions will be made based on a low‐ or high‐risk ODXRS.

Patients with an average modified Magee score of ≤18 are highly likely to have an ODXRS <26. Given the recent TAILORx findings,^35^ we strongly recommend that patients with an average modified Magee score of ≤18 who meet low risk RoMMa histologic criteria not be sent out for ODX testing if the clinician is not considering chemotherapy for an ODXRS <26.

Our results also suggest that the likelihood of breast cancer recurrence in patients who satisfy RoMMA low‐risk criteria is comparable with outcomes in patients with an ODXRS <11, and better than an ODXRS <18.

We suggest a “stepwise” approach when risk stratifying breast cancer patients. One approach might be to use information from the RoMMa or similarly less expensive validated models to help identify cases with already available clinical and pathological metrics that will likely elicit information that is similar to ODX. In these cases ODX may not provide any additional significant clinical utility, and would likely not be cost‐effective or cost‐efficient. ODX testing could then be limited to cases where the assay results would potentially provide clinical utility beyond the available clinical and pathologic metrics. The potential cost savings to the health‐care system would be significant. Support for this “stepwise” approach will require additional validation of the RoMMa in multiple patient cohorts with outcome data to help insure that the information obtained is indeed generalizable to the broader breast cancer population.

## CONFLICT OF INTERESTS

All authors have no conflict of interest.

## AUTHOR CONTRIBUTIONS

Dr Turner: Conceptualization, project administration, data curation, methodology, formal analysis, original draft, writing, review, and editing. Dr Gimenez‐Sanders: Data curation, writing, review, and editing. Mr Soukiazian: Data curation, formal analysis, review. Dr Breaux: Data curation, review, and editing. Dr Skinner: Review and editing. Dr Shayne: Review and editing. Ms Soukaizian: Data curation. Dr Ling: Review. Dr Hicks: Conceptualization, methodology, review, and editing.
