# Supplementary figures and images for: Risk stratification of ER‐positive breast cancer patients: A multi‐institutional validation and outcome study of the Rochester Modified Magee algorithm (RoMMa) and prediction of an Oncotype DX® recurrence score <26
Source: Cancer Med. 2019 Jun 14;8(9):4176–88. doi: 10.1002/cam4.2323 (PMC6675710; doi:10.1002/cam4.2323)

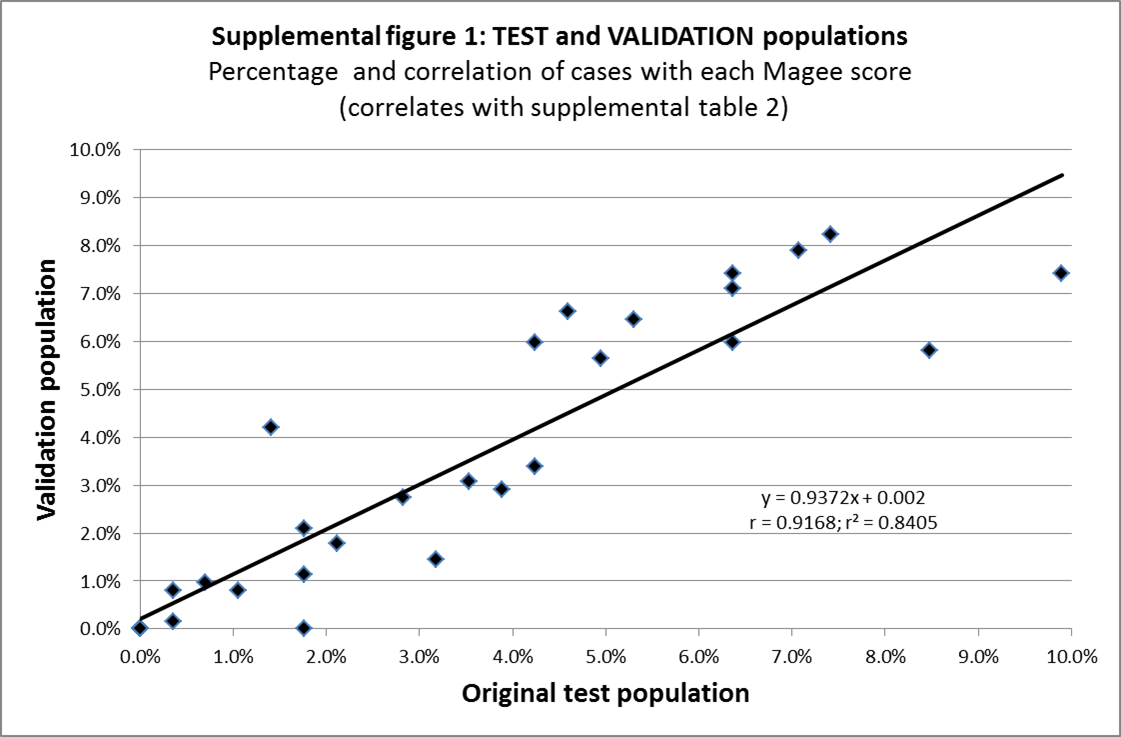

Supplement: Supplementary file 1 [file CAM4-8-4176-s001.tif]

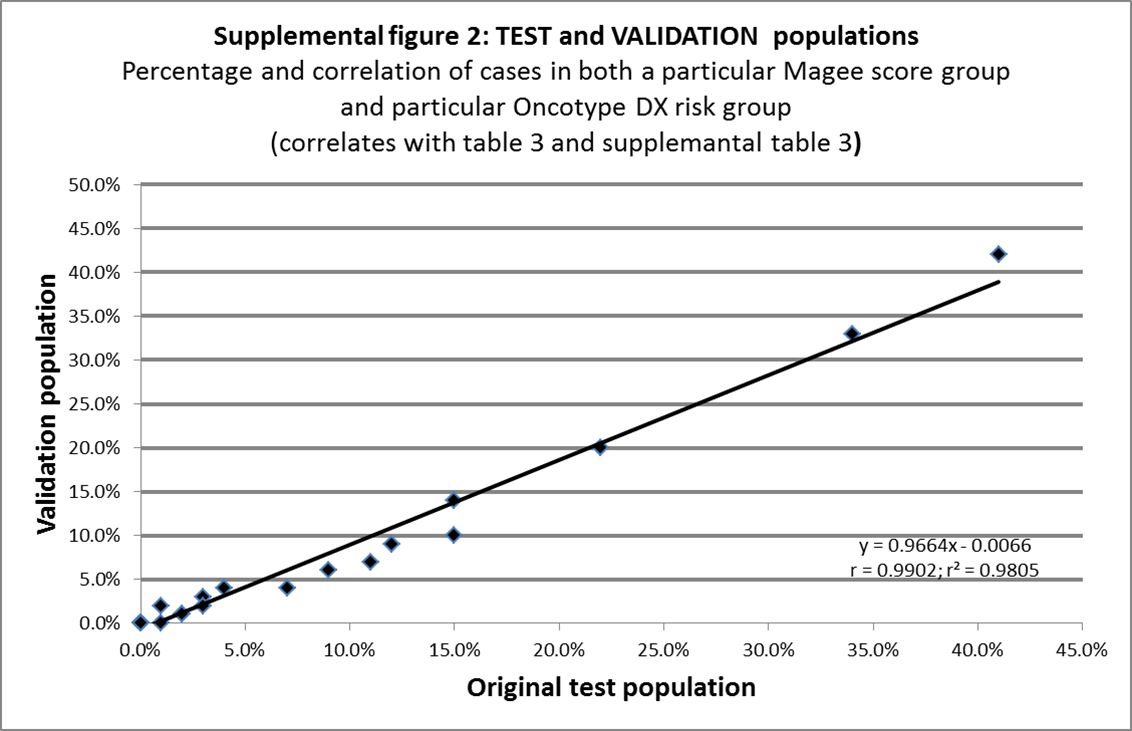

Supplement: Supplementary file 2 [file CAM4-8-4176-s002.tif]
