# Supplementary material for: Risk stratification of ER‐positive breast cancer patients: A multi‐institutional validation and outcome study of the Rochester Modified Magee algorithm (RoMMa) and prediction of an Oncotype DX® recurrence score <26
Source: Cancer Med. 2019 Jun 14;8(9):4176–88. doi: 10.1002/cam4.2323 (PMC6675710; doi:10.1002/cam4.2323)
Supplement: Supplementary file 3 [file CAM4-8-4176-s003.docx]

**APPENDIX WITH SUPPLEMENTAL TABLES**

**Supplemental Table 1A**

**Number of high (>30), intermediate (≥18 and ≤30), and low (<18) average modified Magee recurrence scores and** **Oncotype DX^®^ recurrence scores (n = 620)**

| **Average modified Magee score** | ***Oncotype DX^®^ recurrence score***  ***TOTAL POPULATION*** | | | ***Oncotype DX^®^ recurrence score***  ***University of Rochester*** | | | ***Oncotype DX^®^ recurrence score***  ***University of Louisville*** | | |  |
| --- | --- | --- | --- | --- | --- | --- | --- | --- | --- | --- |
|  | *High* | *Intermediate* | *Low* | *High* | *Intermediate* | *Low* | *High* | *Intermediate* | *Low* | ***TOTAL*** |
| High | 17 | 0 | 0 | 13 | 0 | 0 | 4 | 0 | 0 | **17** |
| Intermediate | 40 | 147 | 91 | 30 | 115 | 77 | 10 | 32 | 14 | **278** |
| Low | 1 | 63 | 261 | 0 | 43 | 191 | 1 | 20 | 70 | **325** |
| **TOTAL** | **58** | **210** | **352** | **43** | **158** | **268** | **15** | **52** | **84** | **620** |

**Supplemental Table 1B**

**Average modified Magee recurrence score groups associated Oncotype DX^®^ risk categories from the validation population by institution (n = 620)**

| **Average modified Magee score (amMs)*** | ***Oncotype DX^®^ recurrence score***  ***University of Rochester*** | | | | ***Oncotype DX^®^ recurrence score***  ***University of Louisville*** | | | |
| --- | --- | --- | --- | --- | --- | --- | --- | --- |
|  | *High* | *Intermediate* | *Low* | ***%***** | *High* | *Intermediate* | *Low* | ***%***** |
| amMs<9 | 0 | 0 | 4 | **100.0%** | 0 | 0 | 0 | **NA***** |
| amMs ≤10 | 0 | 2 | 17 | **89.5%** | 0 | 0 | 5 | **100%** |
| amMs ≤11 | 0 | 6 | 35 | **85.4%** | 0 | 1 | 10 | **90.9%** |
| amMs ≤12 | 0 | 10 | 64 | **86.5%** | 0 | 3 | 20 | **87.0%** |
| amMs ≤14 | 0 | 18 | 124 | **87.3%** | 0 | 10 | 45 | **81.8%** |
| amMs ≤15 | 0 | 23 | 147 | **86.5%** | 1 | 13 | 59 | **81.9%** |
| amMs <18 | 0 | 43 | 191 | **81.6%** | 1 | 20 | 70 | **77.8%** |
| NS<6 and ER/PR≥150 AND Ki67<10% | 0 | 11 | 49 | **81.7%** | 0 | 0 | 7 | **100%** |
| amMs >30 | 13 | 0 | 0 | **100.0%** | 4 | 0 | 0 | **100.0%** |

***** Cases with an available Ki-67

****** Percent Oncotype DX^®^ low risk except for amMs >30 which would be percent Oncotype DX^®^ high risk

*******Not applicable

**Supplemental Table 2**

**TEST and VALIDATION populations:**

**Percentage of cases with each Magee score**

**(correlates with supplemental figure 1)**

| **Average modified Magee score** | **ORIGINAL** | **VALIDATION** |
| --- | --- | --- |
| **≤1** | 0.0% | 0.0% |
| **>1, ≤2** | 0.0% | 0.0% |
| **>2, ≤3** | 0.0% | 0.0% |
| **>3, ≤4** | 0.0% | 0.0% |
| **>4, ≤5** | 0.0% | 0.0% |
| **>5, ≤6** | 0.0% | 0.0% |
| **>6, ≤7** | 0.4% | 0.2% |
| **>7, ≤8** | 1.8% | 0.0% |
| **>8, ≤9** | 1.1% | 0.8% |
| **>9, ≤10** | 3.2% | 1.5% |
| **>10, ≤11** | 4.2% | 3.4% |
| **>11, ≤12** | 4.9% | 5.6% |
| **>12, ≤13** | 7.4% | 8.2% |
| **>13, ≤14** | 7.1% | 7.9% |
| **>14, ≤15** | 9.9% | 7.4% |
| **>15, ≤16** | 6.4% | 7.4% |
| **>16, ≤17** | 6.4% | 7.1% |
| **>17, ≤18** | 8.5% | 5.8% |
| **>18, ≤19** | 4.2% | 6.0% |
| **>19, ≤20** | 6.4% | 6.0% |
| **>20, ≤21** | 4.6% | 6.6% |
| **>21, ≤22** | 5.3% | 6.5% |
| **>22, ≤23** | 1.4% | 4.2% |
| **>23, ≤24** | 3.5% | 3.1% |
| **>24, ≤25** | 3.9% | 2.9% |
| **>25, ≤26** | 2.1% | 1.8% |
| **>26, ≤27** | 1.8% | 2.1% |
| **>27, ≤28** | 1.8% | 1.1% |
| **>28, ≤29** | 0.7% | 1.0% |
| **>29, ≤30** | 0.4% | 0.8% |
| **>30** | 2.8% | 2.7% |

**Supplemental Table 3**

**TEST and VALIDATION populations:**

**Percentage of cases in a particular average modified Magee score (amMs) group and its correlating Oncotype DX risk group (correlates with table 3 and supplemental figure 2)**

| **Magee score group and Oncotype risk group** | **ORIGINAL** | **VALIDATION** |
| --- | --- | --- |
| **Oncotype high risk** |  |  |
| amMs <9 | 0.0% | 0.0% |
| amMs ≤10 | 0.0% | 0.0% |
| amMs ≤11 | 0.0% | 0.0% |
| amMs ≤12 | 0.0% | 0.0% |
| amMs ≤14 | 0.0% | 0.0% |
| amMs ≤15 | 0.0% | 0.0% |
| amMs <18 | 0.0% | 0.0% |
| NS<6 and ER/PR≥150 AND Ki67<10% | 0.0% | 0.0% |
| amMs >30 | 3.0% | 3.0% |
| **Oncotype intermediate risk** |  |  |
| amMs <9 | 0.0% | 0.0% |
| amMs ≤10 | 1.0% | 0.0% |
| amMs ≤11 | 2.0% | 1.0% |
| amMs ≤12 | 3.0% | 2.0% |
| amMs ≤14 | 4.0% | 4.0% |
| amMs ≤15 | 9.0% | 6.0% |
| amMs <18 | 15.0% | 10.0% |
| NS<6 and ER/PR≥150 AND Ki67<10% | 1.0% | 2.0% |
| amMs >30 | 0.0% | 0.0% |
| **Oncotype low risk** |  |  |
| amMs <9 | 2.0% | 1.0% |
| amMs ≤10 | 7.0% | 4.0% |
| amMs ≤11 | 11.0% | 7.0% |
| amMs ≤12 | 15.0% | 14.0% |
| amMs ≤14 | 22.0% | 20.0% |
| amMs ≤15 | 34.0% | 33.0% |
| amMs <18 | 41.0% | 42.0% |
| NS<6 and ER/PR≥150 AND Ki67<10% | 12.0% | 9.0% |
| amMs >30 | 0.0% | 0.0% |

**Supplemental Table 4**

**TEST and VALIDATION populations:**

**Observed frequencies in the original test population and the validation population when evaluating number of cases with an average modified Magee score < 18 or ≥ 18**

|  | Average modified Magee score | |
| --- | --- | --- |
|  | <18 | ≥18 |
| ORIGINAL | **158** | **125** |
| VALIDATION | **325** | **295** |

p = 0.351

**Supplemental Table 5**

**TEST and VALIDATION populations:**

**Observed frequencies in cases with an average modified Magee score (amMs) ≤ 18 in the original test population and the validation population, with an Oncotype DX score < 26 or ≥ 26**

|  | amMs ≤ 18 with ODX* < 26 | amMs ≤ 18 with ODX* ≥ 26 |
| --- | --- | --- |
| ORIGINAL | **175** | **3** |
| VALIDATION | **349** | **10** |

* Oncotype DX score

p = 0.559

**Supplemental Table 6: Outcome cases- Clinical data on recurrent cases**

| **CASE** | **Oncotype DX^®^ recurrence score** | **Average modified Magee score** | **Age** | **Hormone therapy*** | **Chemotherapy**** | **Radiation therapy** | **Nodal status** | **LVI***** | **Alive** | **Nottingham score** | **Modified**  **ER H-score** | **Modified**  **PR H-score** | **Ki-67** | **Follow-up**  **(years)** | **Recurrence type** |
| --- | --- | --- | --- | --- | --- | --- | --- | --- | --- | --- | --- | --- | --- | --- | --- |
| **1** | **5** | **13.5** | **57** | **ANAS** | **NO** | **NO** | **pN0(sn)** | **NO** | **YES** | **6** | **285** | **285** | **NR^ǂ^** | **11** | **Local** |
| **2** | **11** | **19.1** | **60** | **NO** | **NO** | **NO** | **pN0** | **NO** | **YES** | **6** | **240** | **180** | **27** | **7** | **Distant** |
| **3** | **13** | **19.7** | **54** | **ANAS** | **NO** | **YES** | **pN0(sn)** | **NO** | **YES** | **5** | **300** | **15** | **20** | **7** | **Local** |
| **4** | **14** | **21.4** | **61** | **TAM** | **NO** | **YES** | **pN0(sn)** | **YES** | **NO** | **9** | **285** | **285** | **55** | **2** | **Distant** |
| **5** | **15** | **14.0** | **63** | **EXEM /ANAS/TAM** | **NO** | **YES** | **pN0** | **NO** | **NO** | **5** | **270** | **210** | **NR** | **8** | **Distant** |
| **6** | **15** | **21.6** | **59** | **ANAS** | **NO** | **YES** | **pN1a** | **NO** | **YES** | **5** | **120** | **90** | **5** | **3** | **Distant** |
| **7** | **16** | **15.0** | **70** | **ANAS** | **NO** | **NO** | **pN0** | **YES** | **YES** | **5** | **270** | **180** | **5** | **6** | **Distant** |
| **8** | **16** | **6.5** | **64** | **NO** | **NO** | **YES** | **pN0(sn)** | **UNK^ǂǂ^** | **YES** | **3** | **285** | **285** | **NR** | **10** | **Distant** |
| **9** | **17** | **20.2** | **50** | **YES (UNK)** | **YES (UNK)** | **YES** | **pN0(sn)** | **YES** | **YES** | **8** | **255** | **255** | **NR** | **8** | **Local** |
| **10** | **17** | **27.2** | **72** | **ANAS** | **NO** | **NO** | **pN1(mi)** | **YES** | **YES** | **6** | **270** | **1** | **60** | **4** | **Distant** |
| **11** | **19** | **16.3** | **43** | **LUP/TAM** | **NO** | **YES** | **pN0** | **NO** | **YES** | **4** | **285** | **45** | **NR** | **8** | **Distant** |
| **12** | **24** | **24.5** | **51** | **NO** | **CYCL/MET/5-FU** | **NO** | **pN0** | **YES** | **NO** | **8** | **240** | **105** | **35** | **3** | **Distant** |
| **13** | **27** | **23.9** | **21** | **LUP/LET** | **DOC/CYCL** | **YES** | **pN0** | **NO** | **YES** | **7** | **210** | **100** | **35** | **6** | **Distant** |
| **14** | **28** | **21.0** | **68** | **ANAS/EXEM** | **NO** | **NO** | **pN1a** | **NO** | **YES** | **5** | **270** | **21** | **25** | **7** | **Distant** |
| **15** | **28** | **23.4** | **71** | **ANAS** | **NO** | **YES** | **pN1c** | **YES** | **NO** | **5** | **285** | **30** | **35** | **2** | **Distant** |
| **16** | **31** | **28.7** | **81** | **ANAS** | **NO** | **YES** | **pN1a** | **YES** | **NO** | **8** | **270** | **1** | **45** | **2** | **Distant** |
| **17** | **34** | **18.7** | **28** | **YES (UNK)** | **YES (UNK)** | **NO** | **pN0(sn)** | **NO** | **YES** | **7** | **285** | **210** | **35** | **5** | **Distant** |
| **18** | **44** | **32.3** | **83** | **ANAS** | **NO** | **NO** | **pN0** | **NO** | **NO** | **9** | **210** | **60** | **70** | **3** | **Distant** |
| **AVG** | **20.8** | **20.4** | **58.7** |  |  |  |  |  |  | **6.2** | **257.5** | **131.0** | **34.8** | **5.7** |  |

*** ANAS = Anastrozole; EXEM = Exemestane; LET = Letrozole; LUP = Lupron; TAM = Tamoxifen**

**** CYCL = Cyclophosphamide; DOC = Docetaxel; 5-FU = 5-fluorouracil; MET = Methotrexate
*** Lymphovascular invasion**

**ǂ Ki-67 not reported**

**ǂǂ Unknown**

**Supplemental table 7: Oncotype DX^®^ score, Lymph node (LN) status, Lymphovascular invasion (LVI) status and Recurrence**

|  | **RECURRENCE** | | | | | | | | | |
| --- | --- | --- | --- | --- | --- | --- | --- | --- | --- | --- |
|  | **YES** | | | | | **NO** | | | | |
|  | **N** | ***Nottingham score** | ***Modified**  **ER H-score** | ***Modified**  **PR H-score** | ***Ki-67** | **N** | ***Nottingham score** | ***Modified**  **ER H-score** | ***Modified**  **PR H-score** | ***Ki-67** |
| **TOTAL POPULATION** | **18** | **6.2 (3-9)** | **257.5 (120-300)** | **131.0 (1-285)** | **33.4 (5-70)** | **283** | **5.8 (3-9)** | **247.0 (10-300)** | **178.4 (0-300)** | **16.0 (1-90)** |
| **Oncotype Dx score** |  |  |  |  |  |  |  |  |  |  |
| **< 26** | **12** | **5.8 (3-9)** | **258.8 (120-300)** | **161.3 (1-285)** | **27.8 (5-60)** | **246** | **5.5 (3-9)** | **252.7 (55-300)** | **193.8 (0-300)** | **13.1 (1-50)** |
| **≥ 26** | **6** | **6.8 (5-9)** | **255.0 (210-285)** | **70.3 (1-210)** | **40.8 (25-70)** | **37** | **7.5 (5-9)** | **209.4 (10-297)** | **76.4 (0-297)** | **35.6 (5-90)** |
| **LN status** |  |  |  |  |  |  |  |  |  |  |
| **Positive** | **5** | **5.7 (5-8)** | **247.5 (120-285)** | **68.8 (1-270)** | **30.8 (5-60)** | **47** | **5.8 (3-9)** | **248.7 (70-300)** | **183.0 (0-285)** | **14.3 (1-60)** |
| **Negative**** | **13** | **6.3 (3-9)** | **263.1 (210-300)** | **170.4 (15-285)** | **35.3 (5-70)** | **223** | **5.8 (3-9)** | **247.0 (10-300)** | **178.1 (0-300)** | **16.5 (1-90)** |
| **LVI status** |  |  |  |  |  |  |  |  |  |  |
| **Positive** | **7** | **7 (5-9)** | **267.9 (240-285)** | **122.4 (1-285)** | **39.2 (5-60)** | **28** | **6.9 (4-9)** | **240.7 (60-300)** | **156.2 (0-285)** | **18.9 (2-75)** |
| **Negative** | **10** | **5.8 (4-9)** | **249.5 (120-300)** | **135.1 (15-270)** | **29.0 (5-70)** | **248** | **5.7 (3-9)** | **247.9 (10-300)** | **181.3 (0-300)** | **15.6 (1-90)** |
| **Chemotherapy** |  |  |  |  |  |  |  |  |  |  |
| **Yes** | **4** | **7.5 (7-8)** | **247.5(210-285)** | **167.5 (100-255.0)** | **35 (35-35)** | **55** | **6.8 (3-9)** | **224.3 (10-300)** | **135.8 (0-285)** | **24.5 (1-80)** |
| **No** | **14** | **5.8 (3-9)** | **260.0 (120-300)** | **120.6 (1-285)** | **32.9 (5-70)** | **176** | **5.3 (3-9)** | **252.4 (50-300)** | **186.1 (0-300)** | **12.6 (1-60)** |

*** Average (range)**

**** Includes isolated tumor cells (n = 7)**
